# Exploring antioxidant potential of agricultural by-products: a systematic review

**DOI:** 10.12688/f1000research.145702.1

**Published:** 2024-09-04

**Authors:** Imam Santoso, Suprayogi Suprayogi, Akhmad Adi Sulianto, Endrika Widyastuti, Annisa’U Choirun, Khairunnisa Lestari, Syairil A’yuniah, Octavia Widyastuti Kusumaningtyas

**Affiliations:** 1 Agroindustrial Technology, Brawijaya University, Malang, East Java, 65145, Indonesia; 2 Biosystem Engineering, Brawijaya University, Malang, East Java, 65145, Indonesia; 3 Food Science and Biotechnology, Brawijaya University, Malang, East Java, 65145, Indonesia; 4 Agricultural Technology, Politeknik Negeri Jember, Jember, East Java, 68121, Indonesia

**Keywords:** antioxidant activity, extraction, agricultural by-product

## Abstract

**Background:**

Agricultural waste sourced from various activities that occur along the agricultural supply chain including post-harvest, processing, and consumption processes, can pose a threat to ecosystem balance and community welfare. Data shows that agricultural by-products have the potential to be utilized because they contain antioxidant compounds. This systematic review study aims to identify and assess the antioxidant activity of agricultural by-products through various extraction methods.

**Methods:**

This systematic review collected literature in the last 10 years (2013–2023) from Google Scholar, Semantic, and Scopus-indexed articles with the help of Publish or Perish. Using the help of boolean operators (AND) and (OR) in searching using keywords. The steps applied adapt the PRISMA method (Preferred Reporting Items for Systematic Reviews and Meta-Analyses), including identification, screening, eligibility, and inclusion.

**Results:**

Literature collection data shows that the dominant processing method used is the solvent extraction method to determine the antioxidant value of various agricultural waste by-products. Followed by microwave-assisted extraction (MAE) and ultrasound-assisted extraction (UAE) methods. A wide range of antioxidant activity values were found depending on the type of agricultural waste and processing technique. One potential utilization of agricultural wastes rich in antioxidant content is as additives in formulations in the cosmetic industry.

**Conclusion:**

Agricultural waste by-products have high potential of antioxidant content, depending on the type of waste and extraction method. The dominant agricultural waste used is by-products from the fruit group. The utilization of agricultural waste that is rich in antioxidants has the potential to be utilized in the cosmetic industry.

## Introduction

The rapid growth of the agricultural sector through increased production and processing of agricultural products in recent decades has had a significant impact on the environment. Between 2000 and 2020, world production of major food crops increased by 52% to 9.3 billion tons, which is equivalent to 2019 production. Vegetables represent 20%, followed by fruits at 17%, and root crops at 7%. ^1^ A significant amount of agricultural waste is sourced from fruits, nuts, and vegetables resulting from the various activities that occur along the agricultural supply chain, which include post-harvest processing, processing, and consumption. ^2^^,^^3^ The agricultural sector accounted for 31% of total greenhouse gas emissions during the period 2000–2020. ^1^ China will be one of the largest greenhouse gas emitters in 2022, accounting for 50.1% of the global population and 63.4% of global fossil fuel consumption. ^4^ In addition, more than half of the total waste distribution comes from fruits and vegetables, while rice and wheat also contribute significantly and are expected to increase to 234 Mt by 2032. ^1^

Agricultural waste contains various organic materials, carbohydrates, and other minerals that, if allowed to accumulate, can serve as a growing medium for decaying microorganisms. The significant increase in waste volume poses a threat to the balance of the ecosystem and the well-being of society. In recent years, a number of studies have indicated that agri-food residues contain a number of valuable compounds with potential bioactivity. ^5^^–^^8^ Therefore, agricultural waste can serve as a valuable renewable resource, offering additional benefits such as affordability, easy accessibility, minimal environmental impact, and sustainability. The antioxidant compounds contained in the waste have the potential to be used as food supplements or medicines, neutralizing free radicals and reducing their adverse effects on human health. ^9^^–^^11^ In addition, it can be utilized as a raw material for the production of other products, such as biofuels, biogas, bioethanol, animal feed, and composting. ^12^^–^^16^

The waste extraction process can open up opportunities to obtain these compounds efficiently and sustainably. ^17^ In addition, the extraction and use of antioxidants from agricultural waste can reduce environmental impact, reduce the amount of organic waste, and add value to the waste. This utilization not only provides a solution to the waste problem, but also opens up new business opportunities, reduces the need for new raw materials, and supports a sustainable economic approach. With increasing awareness of the economic and environmental value of agricultural waste, it is necessary to develop environmentally friendly and sustainable alternatives to conventional extraction methods to increase extraction yield and reduce extraction time and solvent consumption. To understand more about the utilization of antioxidant content in agro-industrial waste, further research and analysis of scientific articles and publications in the field of agricultural waste management and utilization can be conducted. More and more studies are reviewing the utilization of agricultural by-products and the potential content of organic compounds in agricultural waste. ^18^ This extensive knowledge suggests that the antioxidant content of agricultural wastes is a promising alternative for supporting sustainable development.

The aim of this systematic review was to identify and assess all findings in articles related to the antioxidant activity of agricultural by-products. The extraction technologies used are briefly presented. Ultimately, this paper will provide insights for future research on the potential utilization of antioxidant content from agricultural by-products under the concept of sustainability.

## Methods

### Planning

The writing of this systematic literature review is based on the PRISMA (Preferred Reporting Items for Systematic Reviews and Meta-Analysis) method. ^19^ Article data will be used in this systematic review if it meets the following criteria: 1) Samples are agricultural waste (except animals); 2) Analyze the antioxidant activity using the extraction method; 3) The article is a research article; 4) Articles included in this selection have been published within the last 10 years (2013–2023). Studies showing animal samples and belonging to the type of review article or conference were excluded.

### Search approach

The database in the systematic review used Google Scholar, Semantic, and scopus-indexed articles with the help of an electronic database search, namely Publish or Perish. The literature search was limited to the year of publication between 2013 and 2023. Boolean operators (AND, OR) were used to combine keywords in order to identify relevant articles. Search terms (“agricultural by-product” OR “agricultural waste”) AND “extraction” AND (“antioxidant” OR “antioxidant activity”) to find related studies and data.

### Data extraction

Duplicate identification was carried out using Mendeley by importing all reference lists that had been obtained, and then duplicate data was deleted. Next, a screening process was carried out based on the title and abstract. Articles that passed entered the next stage, namely full-text screening to identify the type of waste sample discussed, the extraction method, and the antioxidant activity produced. The VOS Viewer generates a visual display of bibliographic materials using custom input data. The search terms represent the inclusion criteria and are closely linked to the study’s objectives, scope, limitations, and areas for further exploration. Moreover, only English-language original articles will be taken into account.

## Result

### Study selection result

**Figure 1.**
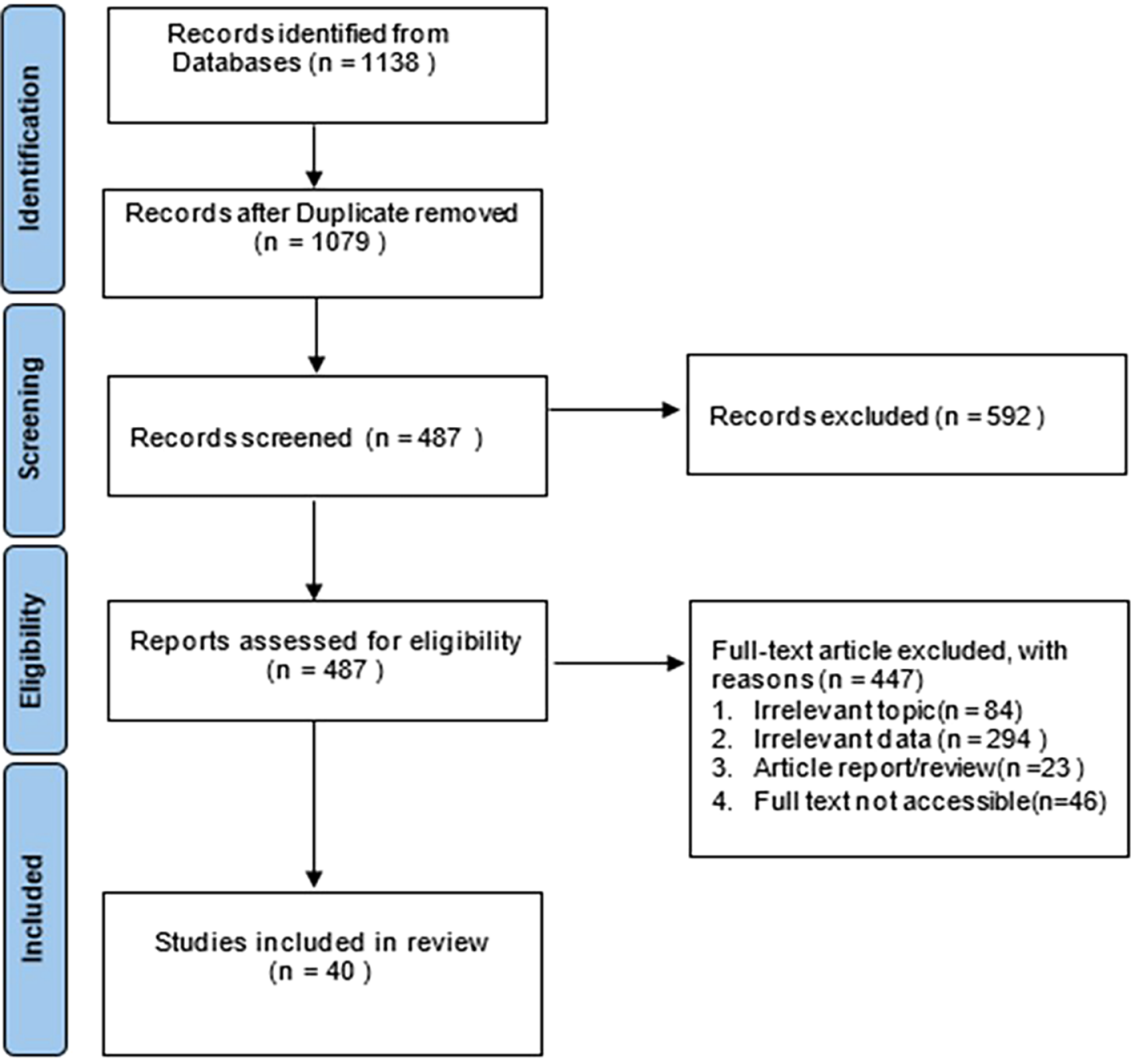
PRISMA flow chart for literature search.

The article search conducted on the database mentioned in Figure 1, with the help of keywords and boolean operators obtained 1138 articles. After 59 duplicates were identified, 1079 articles were screened for title and abstract, and 487 articles were considered potentially eligible. A second screening was conducted, leaving 40 final articles for analysis.

**Table 1.** Antioxidant activity of agricultural by-products (23-62).

Year	By-Product	Extraction Technology (Process)	Antioxidant Activity	Reference
2019	Red, white grape pomase and canes	Heat reflux (solid - liquid) extraction	37.5-262.5 μg/mL ^(a)^	^23^
2019	Pressed canola meal	Accelerated solvent extraction (ASE) with ethanol* and methanol**)	*9.64-12.86 mg SAE/g DM ^(a)^; **10.13-24.71 mg SAE/g DM ^(a)^; 0.383 to 0.405 mmol TE/g DM ^(c)^	^24^
2022	Oilseed meal/and sesame seeds	Conventional with ethanol solvent, UAE with ethanol and distilled water	58.5 ± 0.6-65.63 ± 1.4 (g TE/100 g ^(a)^; 2.42 ± 0.01-2.77 ± 0.02 (g TE/100 g) ^(c)^	^25^
2023	The leaves of X. sorbifolia	Conventional and (UAE) using ethanol	29.89 ±1.80-92.46±0.87% ^(a)^; 35.14±0.14-94.90±0.36% ^(b)^	^26^
2022	Bael pulp residue	Hot acid extraction with, 1 M HCl and 1 M H _2_SO _4_	15.74 μg/mL ^(a)^	^27^
2022	Red grape stalk	Supercritical fluid extraction (SFE) and ultrasound extraction	2.96 ± 0.139-22.2 ± 2.828 mg Trolox/g ^(a)^; 61.38 ± 0.469-108.29 ± 10.811 mg Trolox/g ^(b)^	^28^
2021	Soybean hull ( *Glycine max*)	Conventional extraction with organic and alkaline solvent	0.516 ± 0.002 mmol TE/100 g SH ^(b)^	^29^
2022	Walnut shells* and walnut green husk**	Solvent extraction with ethanol, methanol, acetone	*21.2 ± 1.65 mg TE/g - 28.74 ± 2.16 mg TE/g ^(a)^; *82.5-3 mg GAE/g	^30^
2022	Kabkab date seed ( *Phoenix dactylifera* L.)	Microwave - assisted extraction (MAE)*, Ultrasound - assisted extraction (UAE)**,	*1.04 ± 0.12-1.19 ± 0.15 mg mL ^−1^; 0.77 ± 0.01 - **0.79 ± 0.02 mg mL ^−1^	^31^
2022	Papaya pulp*and peel**	Pulsed ultrasound-assisted extraction (PUSAE) and Hot water extraction (HWEX)	*31.29 ± 0.37-87.17 ± 0.26; **97.36 ± 0.24-292.2 ± 0.12 %	^32^
2022	Cacao pod husk (CPH)	Maseration, Microwave assisted extraction (MAE): with ethanol/water, Conventional solvent extraction (CSE)	3.36 ± 0.02 mg TE/g dw ^(a)^	^33^
2022	Avocado peel	Solid - liquid extraction	37.30 ± 1.00-93.92 ± 1.29% ^(a)^	^34^
2021	Pistachio hull	Conventional solvent extraction with water, ethanol, methanol, n-hexane, and acetone	42.6-100% ^(a)^	^35^
2021	Custard apple seeds	Supercritical fluid extraction (SFE)− CO _2_	0.08 ± 0.11-0.39 ± 0.09 mg/g ^(a)^	^36^
2018	Husk of Coconut (mesocarp and exocarp)	Microwave assisted extraction with 0.1 M NaOH solvent	(119.96 mM TE/g) (Mesocarp), 55.27 mM TE/g (Exocarp) ^(a)^	^37^
2023	cocoa ( *Theobroma cacao *L.) leaves	The extraction applied maceration method using methanol solvent	TPC = 349.826 mg EAG/g sample ^(a)^, 86.928% RSA ^(b)^, EC50 = 287.958 ppm ^(c)^	^38^
2020	Coffee Pulp	Fermentation process with lactic acid bacteria, followed by methanol solvent maceration extraction.	27.6% at 100 ppm ^(a)^	^39^
2020	walnut shell	Ultrasound Assisted Extraction (UAE)	4.05-88.59% at (10-500 μg/mL) ^(a)^; 119.64-1278.95 μM TE/g at (10-100 μg/mL) ^(b)^	^40^
2016	Banana Peel	Homogenizer-Assisted Extraction (HAE), ethanol	2.44 g GAE/100 g dw ^(a)^; IC50 = 71.74 (mg/mL) ^(b)^	^41^
2014	Grape seeds	Microwave-assisted aqueous two-phase extraction (MAATPE)	IC50= 50.3 mgL ^−1^ ^(a)^; IC50 = 73.1 mgL ^−1^ ^(b)^	^42^
2014	Garlic husk	Extraction using solvents 50/50 methanol–water (v/v, MW)	IC50 = 0.26 mg/mL ^(a)^; RP0.5AU = 2.8 mg/mL ^(b)^, IC50 = 0.45 mg/mL ^(c)^	^43^
2015	Rambutan Peel	Ultrasonic extraction (solvent ration 1: 15 using 40% ethanol, 2 min)	41.15 μmol Fe2+/g DW ^(c)^	^44^
2018	Carrot Waste	Ultrasonic bath extraction (40 min), followed by shaker incubator extraction (120 min)	24.28 % at 40 ppm ^(a)^	^45^
2018	Canvedish Banana Peel	Extraction using ethanol solvent, orbital shaker for 48 hours	IC50 = 90.28 μg/ml ^(a)^	^46^
2020	Lemon Waste	EHD (Electrohydrodynamic) extraction at EHD and EHD voltage	89.76 % at 200 ppm (10 min, 19 kV) ^(a)^	^47^
2020	Coconut endocarp waste	Ultrasound-assisted extraction (UAE)	IC50 = 288.17μg/mL ^(a)^; IC50 = 10.55 μg/mL ^(b)^	^48^
2020	Black mulberry pomace (BMP)	Microwave assisted extraction (MAE), 700 W,300 s, pH1.42 and LSR of 20 mL/g	IC50=63.76μg/mL ^(a)^; IC50 = 46.61 μg/mL ^(b)^	^49^
2019	Ginger pulp and peel	Subcritical water extraction (SWE), 190 °C/15 min (Ginger peel), 190 °C/35 min (Ginger pulp)	6.43 mg TE/g pulp ^(c)^; 4.11 mg TE/g peel ^(c)^	^50^
2020	Pomegranate peel	Vacuum Microwave-Assisted Extraction (VMAE)	IC50 = 1.81 L/min; 5.542 mg (GAE)/g ^(a)^	^51^
2020	Broccoli leaves and stems	Supercritical Fluid Extraction (SFE)	338.69 ± 31.95 mg TE/g ^(a)^	^52^
2020	Potato waste	Heating-assisted extraction at 40 C/30min	1500-1650 μM TE/g. (each different potato fraction) ^(d)^	^53^
2020	Coffee pulp	Microwave−assisted extraction (MAE) with ethanol	4.38 ± 0.01 mg AAE/mL ^(c)^; 97.62 ± 0.26% ^(a)^	^54^
2019	grape juice pomace* and grape wine pomace**	Solid luquid extraction using 40 and 60 temperature; 15 and 45 time, Ethanol and aceton solvent	*EC50 = 349.03 μg m/L ^(a)^; 334.54 μmol TEAC/gμ ^(b)^; 150.94 μmol Fe2+ /g ^(c)^ **EC50 = 488.92 μg m/L ^(a)^; 249.64 μmol TEAC/gμ ^(b)^; 110.73 μmol Fe2+/g ^(c)^	^55^
2018	Pistachio hull ( *Pistacia vera* L.)	Microwave−assisted extraction (MAE) using different ratio ethanol and water solvent	IC50 = 0.70 ± 0.04 mg/mL ^(a)^; 260.86 ± 6.01 μmol TE/g ^(d)^	^56^
2021	Kinnow mandarin peels ( *Citrus reticulata)*	Ultrasonic assited extraction (UAE)	64.70 % ^(a)^; 28.17 mM/100 g ^(c)^; 260.86 ± 6.01 μmol TE/g ^(d)^	^57^
2021	Pineapple peel *(Ananas cosmosus)*	Solid liquid extraction	91.79 ± 1.98 μmol Trolox/g ^(a)^; 174.50 ± 9.98 μmol Trolox/g ^(c)^	^58^
2017	Dragon fruit peels	Hot water exctraction (HWE)	IC50 = 0.0063 mg/mL ^(a)^	^59^
2018	Orange peel *(Citrus sinensis)*	Conventional solvent extraction (CSE) using variation concentration methanol, ethanol, and acetone solvent	8.35-18.20 mg TE/g ^(a)^; 95.00-296.61 mmol FE (II)/g ^(c)^; -0.92 mol TE/g ^(d)^	^60^
2019	Jackfruit peels	Ultrasound microwave-assisted extraction (UMAE)	162.36 ± 10.26 mg TE/g ^(a)^	^61^
2020	Cocoa bean shell ( *Theobroma cacao* L.)	deep eutectic solvents (DESs) coupled with microwave extraction (MAE)	11.751-55.444 % ^(a)^	^62^

^(a)^ 2,2-diphenyl-1-picrylhydrazyl (DPPH) assay.

^(b)^ 2,2′-azino-bis(3-ethylbenzothiazoline-6-sulfonic acid (ABTS) assay.

^(c)^ Ferric reducing antioxidant power (FRAP) assay.

^(d)^ Oxygen radical absorbance capacity (ORAC) assay.

### Article overview

**Figure 2.**
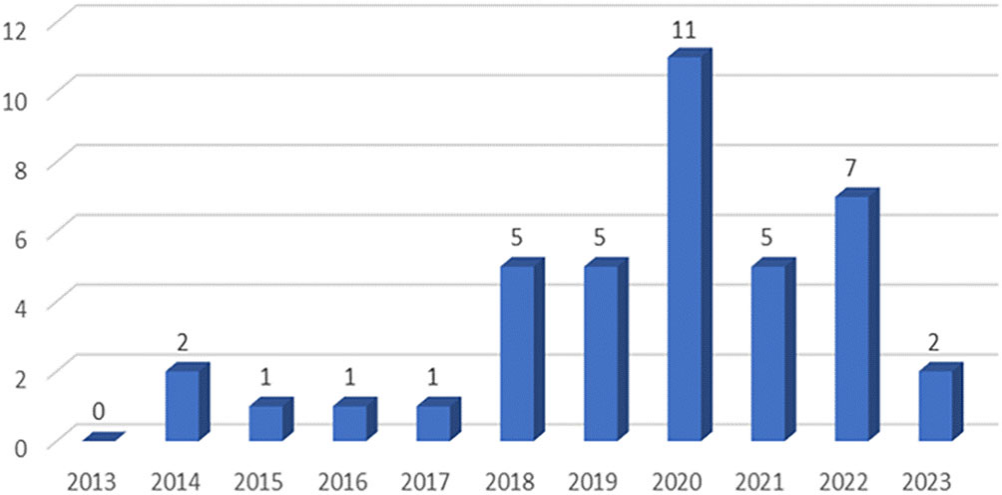
Trend of journal publications.

Figure 2 shows that in the last 10 years, publications on the research of antioxidant potentials from agricultural waste or by-products have fluctuated. In the first half of the decade, between 2013 and 2018 the number of publications tended to be constant at a value of 1–2 publications but there was a significant increase in 2018 reaching 5 publications. The increased number of publications showed a constant value in 2019. Afterwards, there was a sharp increase of 120% from 5 publication articles last year to 11 publication articles in 2020. Nevertheless, for the rest of the second half of the decade between 2021 and 2023, the number of article publications decreased by 2–7 articles per year.

**Figure 3.**
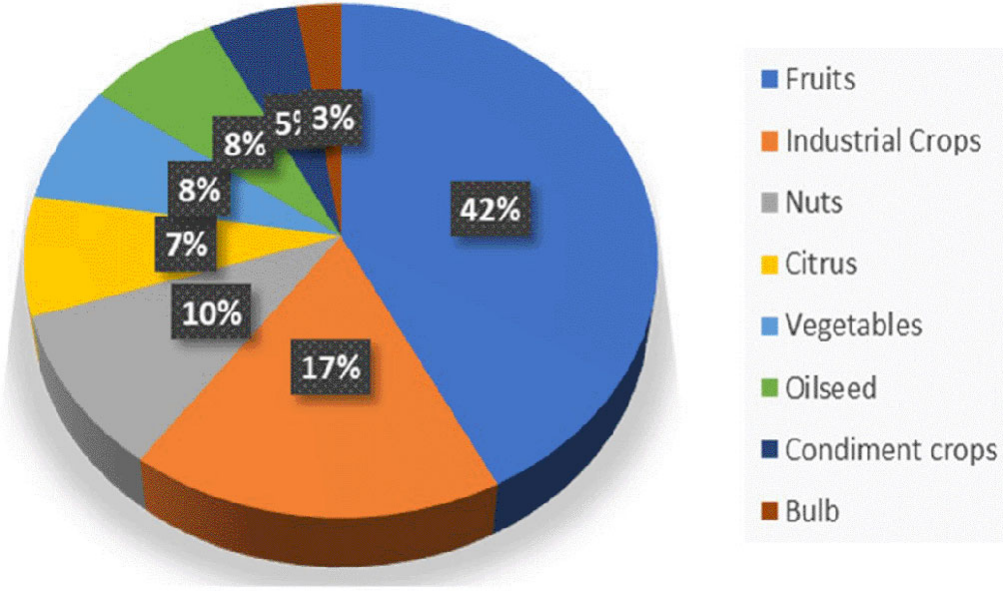
Pie chart of publications by-products.

It is known through Figure 3 that most publication articles are dominated by discussions of processing or extraction of fruit by-products including grape, rambutan, banana, apple, pomegranate, etc followed by industrial crops by-products such as cocoa, coffee, and coconut. While the lowest percentage of research scores are owned by the bulb category which refers to research on the antioxidant potential of garlic. Those are known as parts of agricultural and agro-industrial waste. The classification of by-product types in this systematic review itself is done based on food guide pyramids ^20^ and classification guide of agronomic crops based on its use values. ^21^ Based on trends in publication topics, it is necessary to note that the data used does not include all publication articles published in 2023.

### Keyword analysis

**Figure 4.**
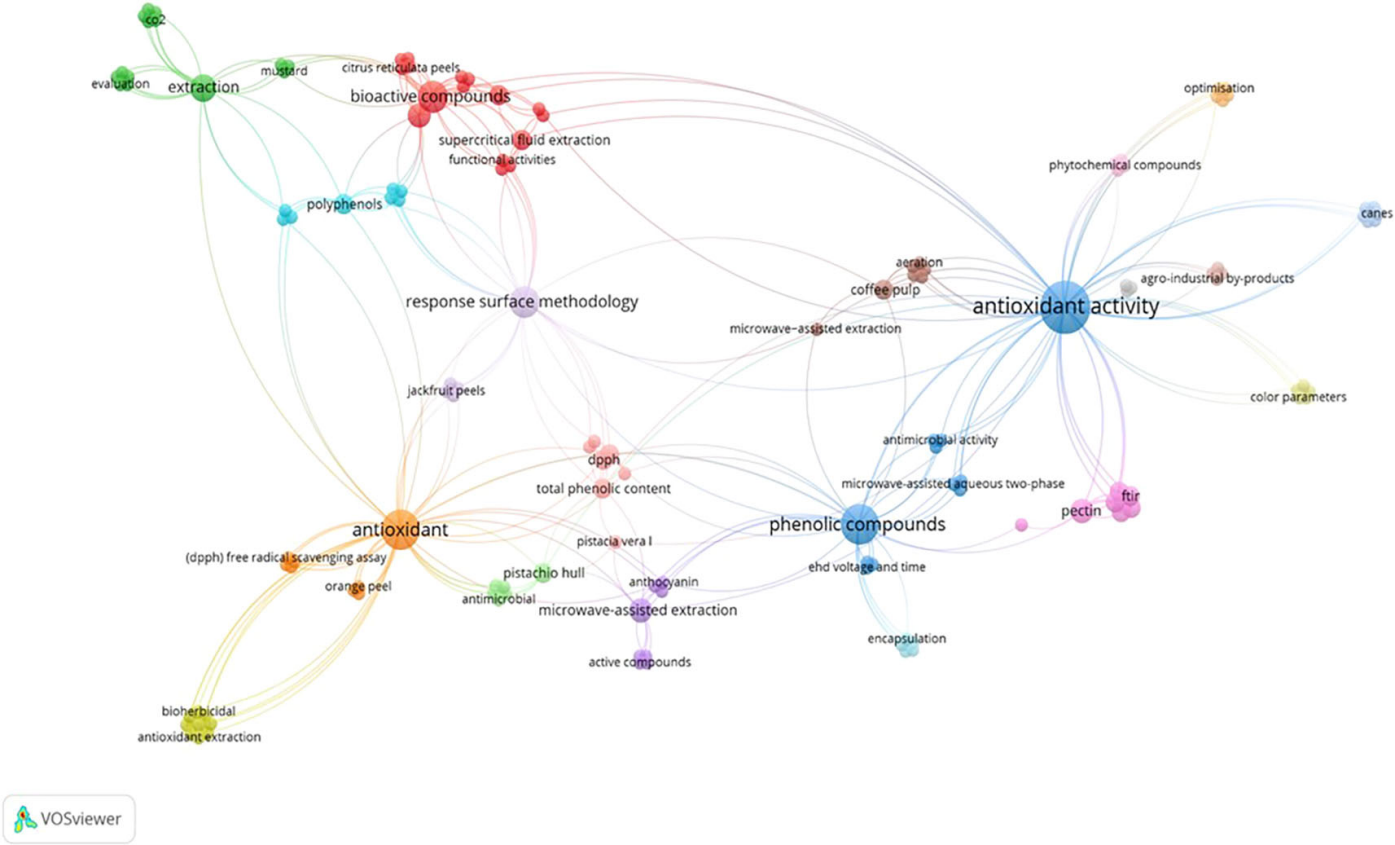
Visualization of frequency used keywords.

Figure 4 is presented about the data extraction results of 40 publication articles obtained with the help of VOSviewer software. The network’s visualization results show that the most common keywords are antioxidants and activity, phenolic compounds, extractions, bioactive compounds, and antioxidants. This is shown by the size of the node as representation of keyword, that is increasing in proportion to the number of publication articles using similar keywords. ^22^ VOSviewer-processed network visualization also shows a link between one keyword and another with a link between nodes. It is known that the most common publication article discussion trends are antioxidant analysis and activities related to acquisition methods such as extraction. It can also be seen that several widely used extraction methods include supercritical fluid extraction (SFE) and microwave-assisted extraction (MAE). Besides the process of obtaining bioactive components, it also can be easily identified from Figure 4 the type of raw material, in which case it is agro-local or agricultural by-product specifically such as coffee pulp, jackfruit peels, pistachio hull, etc.

Furthermore, it is known that there is an optimization keyword trend arising from the processing of publication article data. It generally refers to a publication article that discusses the optimization of the anti-oxidant production process of agricultural waste or by-products itself. In Figure 4. it is known that the optimization method used by it is the response surface methodology. As a whole, it is also known that in the publication article data used, the discussion is not limited to the antioxidant activity of by-product extraction. It also discusses the characteristics of the extract and its application to derivative products, as is known in the presence of the color parameters, functional activities, antimicrobial activity, etc keywords. In addition, data extraction using this VOSviewer essentially facilitates the data analysis process as the connecting lines between nodes also indicate the relationship or interaction of each keyword itself.

Most research make use of underutilised agricultural byproduct peel. ^32^^,^^34^^,^^41^^,^^44^^,^^46^^,^^50^^,^^51^^,^^53^^,^^57^^,^^58^^,^^59^^–^^61^ The extraction methods used varied in each study, mostly using Conventional Solvent Extraction (CSE), ^32^^,^^33^^,^^34^^,^^41^^,^^44^^,^^46^^,^^50^^,^^51^^,^^53^^,^^57^^,^^58^^–^^61^ followed by Microwave - assisted extraction (MAE) ^31^^,^^33^^,^^37^^,^^49^^,^^54^^,^^56^^,^^62^ and Ultrasound-assisted extraction (UAE). ^26^^,^^31^^,^^44^^,^^45^^,^^57^ Other extraction methods used include solid-liquid. ^23^^,^^34^^,^^55^ Accelerated Solvent Extraction (ASE), ^24^ Hot Acid, ^27^ Supercritical fluid extraction (SFE), ^28^^,^^52^ Homogenizer-Assisted Extraction (HAE), ^41^ Maceration. ^33^^,^^38^^,^^39^ Ultrasound - Microwave assisted extraction (UMAE). ^31^^,^^61^ Pulsed ultrasound-assisted (PUSAE). ^32^ Hot water extraction (HWEX), ^32^ Microwave-assisted aqueous two-phase extraction (MAATPE), ^42^ Electrohydrodynamic extraction (EHD), ^47^ Subcritical water extraction (SWE), ^50^ Vacuum Microwave-Assisted Extraction (VMAE), ^51^ DESs. ^62^

**Antioxidant Properties by Agriculture By-products**

The antioxidant activities have been examined differently in each study. Most of study using (2,2-diphenyl-1-picryl-hydrazyl-hydrate) DPPH (Tabel 1.). Based on DPPH assay, lowest IC _50_ in this study is 0,0063 mg/mL in dragon fruit peels. ^59^ However, comparing these data is challenging because other research did not report IC _50_ and utilised different techniques. Other antioxidant assays included (3-ethylbenzothiazolin-6-sulfonic) (ABTS) ^26^^,^^28^^,^^29^^,^^31^^,^^42^^,^^48^^,^^49^^,^^55^; Ferric Reducing Antioxidant Powe (FRAP), ^23^^,^^25^^,^^60^^,^^31^^,^^38^^,^^40^^,^^50^^,^^54^^,^^55^^,^^57^^,^^58^ Radical Absorbance Capacity (ORAC), ^53^^,^^57^^,^^60^ Folin-Ciocalteu. ^30^^–^^33^^,^^51^

## Discussion

Consumption of products rich in bioactive compounds is excellent when it comes to health benefits. Large amounts of by-products during the processing of agricultural raw materials and are suitable starting materials for the extraction of compounds such as natural antioxidants. Phenolic chemicals have been discovered in industrial waste, such as in industries that use grapes as raw material. ^23^^,^^28^^,^^42^^,^^55^ Reported in grape pomace, ^23^^,^^55^ stalk, ^28^ seed, ^42^ is a by-product that contains high anti-oxidant. ^55^ Comparing antioxidant phenolic compounds in grape juice pomace and grape wine pomace using solid luquid extraction, and revealed higher antioxidant capacity in grape juice pomace. In another study, high antioxidant content was also found in fruit peel ^41^^,^^44^^,^^46^^,^^57^^,^^59^^,^^60^ and legume by-products, ^29^^,^^30^^,^^33^^,^^40^^,^^54^^,^^62^ this was shown by the DPPH, ABTS, and FRAP assays. ^63^

According to certain research, the level of antioxidant activity in agricultural waste may be measured using the DPPH (2,2-diphenyl-1-picrylhydrazyl) method. IC50 Value inhibits free radicals by 50% in unit (g/mL or ppm), vich is IC50 inversely proportional with antioxidant activity. The greater the IC50 value, the greater the antiocidant activity of extract (Özbek, 2018). Specifically, a compound is considered to have high antioxidant power if the IC50 value is less than 50 ppm, has moderate power if the IC50 is in the range of 50-100 ppm, and is considered weak when the IC50 value is between 100-150 ppm, and if the IC50 reaches 151-200 ppm. ^64^^,^^65^ From the literature data found, it can be seen that the antioxidant value produced from black mulberry pomace (BMP) waste is 63.76 μg/mL which can be categorized as a strong antioxidant. Meanwhile, the antioxidant activity of coconut endocarp waste is considered to have weak antioxidant activity because the IC50 value is > 200 ppm. Some of the antioxidant tests of the extract materials used standard antioxidant samples in the form of ascorbic acid ^49^^,^^56^^,^^60^ which aims as a comparison. Ascorbic acid was chosen as a comparator because it is characterized by acidity, functions as a highly effective reducer, exhibits high antioxidant activity, and is more polar when compared to other types of vitamin C. ^66^

Antioxidant activities could be affected not only by the types of by-products, but also the extraction method. A lot of studies used a conventional extraction methods, wich is maceration, digestion, infusion and Soxhlet extraction, etc. due to simplicity. Comparison of extractions was done in several studies, such as CSA with UAE ^25^; CSA with DIE, MAE, UAE, UMAE ^31^; and CSA with SFE, ^52^ states that antioxidant activity in extracts obtained by conventional extraction are not higher than those using other methods. The decrease in antioxidant activity is probably because conventional extraction has several weaknesses such as long extraction time and large solvent consumption. ^60^ Therefore, to overcome these problems, several studies have combined conventional extraction with other method. ^26^ Water has been a popular solvent, with certain research showing that mixing water ethano ^54^^,^^60^ by a particular ratio. A study ^56^ mixing etanol-water have the best antioxidant activity for 40% ethanol, another study ^43^ mixing methanol-water the best antioxidant activity with 50% methanol. This shows that the highest antioxidant is obtained in extraction with equal or lower ratio in less polar solvents. ^56^

As a continuation of high antioxidant activity from the discovery of the optimal point of agricultural by-product extraction, antioxidants that are essentially low-concentration substances with the function of delaying or restraining the oxidation of a substrate ^67^ are generally used as additiv materials. In relation, one of the most potential application or use of additional antioxidants is on cosmetic products. In the cosmological industry that is generally related to topical products, the presence of antioxidants in preparation formulations such as cream and so on is expected to help protect the skin through its characteristics that can withstand oxidative stress. ^68^ The addition of antioxidants to cosmic products is considered capable of increasing the value of the product. Where, not only in applications to topical types of products, agricultural by-product extracts with high antioxidant activity can also be formulated in dietary supplements for skin health. ^69^

## Conclusion

Agricultural waste by-products have high potential of antioxidant content, depending on the type of waste and extraction method. The utilization of agricultural waste that is rich in antioxidants has the potential to be utilized in the cosmetic industry. The dominant agricultural waste used is by-products from the fruit group. Antioxidant activity testing generally uses DPPH, ABTS, and FRAP assays. The most widely applied extraction method in agricultural waste processing is solvent extraction, followed by microwave-assisted extraction (MAE) and ultrasound-assisted extraction (UAE).

## Ethics statement

Not required.

## Data Availability

The article includes all the data supporting the results, and there is no need for additional source data. Figshare: PRISMA checklist for: “Exploring antioxidant potential of agricultural by-products: a systematic review”. DOI: https://.doi.org/10.6084/m9.figshare.24715674 Data are available under the terms of the Creative Commons Attribution 4.0 International license (CC-BY 4.0).
