# Supplementary material for: Exploring antioxidant potential of agricultural by-products: a systematic review
Source: F1000Res. 2024 Sep 4;13:1008. [Version 1] doi: 10.12688/f1000research.145702.1 (PMC11474157; doi:10.12688/f1000research.145702.1)
Supplement: Peer review history

# Reviewer report: Reviewer response for version 1

**DOI:** 10.5256/f1000research.159684.r320967

**Published:** 2024-10-14

**Authors:** Emil Salim

**Affiliations:** 1 Department of Pharmacology, Faculty of Pharmacy, Universitas Sumatera Utara, Medan, North Sumatra, Indonesia

The paper is comprehensive in its exploration of antioxidant potential and includes detailed descriptions of various extraction methods.

 The paper clearly outlines the goal of exploring the antioxidant potential of agricultural by-products.

 The methodology follows the PRISMA guidelines and provides clear details on the search strategy, inclusion criteria, and data extraction processes, allowing replication.

 The conclusions align with the review’s findings.

 However, there are minor revision to be made:

 1. Please make the legend of the Y axis in figure 2.

 2. "Water has been a popular solvent, with certain research showing that mixing water ethano ^54^^,^^60^ by a particular ratio. A study ^56^ mixing etanol-water have the best antioxidant activity for 40% ethanol, another study ^43^ mixing methanol-water the best antioxidant activity with 50% methanol. This shows that the highest antioxidant is obtained in extraction with equal or lower ratio in less polar solvents. ^56^", they should be ethanol.

Are the rationale for, and objectives of, the Systematic Review clearly stated?

Yes

Is the statistical analysis and its interpretation appropriate?

Not applicable

If this is a Living Systematic Review, is the ‘living’ method appropriate and is the search schedule clearly defined and justified? (‘Living Systematic Review’ or a variation of this term should be included in the title.)

Not applicable

Are sufficient details of the methods and analysis provided to allow replication by others?

Yes

Are the conclusions drawn adequately supported by the results presented in the review?

Yes

Reviewer Expertise:

Immunology, Pharmacology, natural products.

I confirm that I have read this submission and believe that I have an appropriate level of expertise to confirm that it is of an acceptable scientific standard.

# Reviewer report: Reviewer response for version 1

**DOI:** 10.5256/f1000research.159684.r320969

**Published:** 2024-10-07

**Authors:** Amal Hathout

**Affiliations:** 1 Food Toxicology and Contaminants Department, National Research Centre, Cairo, Egypt

This systematic review aimed to identify and assess the antioxidant activity of agricultural by-products using various extraction methods. Santoso et al.'s study, Exploring the Antioxidant Potential of Agricultural By-products: A Systematic Review, makes an important contribution to the subject of agricultural by-product utilization. The authors effectively address the study topic and present persuasive data to support their findings. The methodology is sound, and the results are noteworthy. The article is well-written and highlights an important issue—the accumulation of agricultural by-products, which poses a threat to the environment.

 Some minor comments that require attention:

1. **Abstract**: Should be shortened.
2. **Introduction**: References should be added to some of the statements. For example:
  - "Between 2000 and 2020, world production of major food crops increased by 52% to 9.3 billion tons, which is equivalent to 2019 production."
  - "Agricultural waste contains various organic materials, carbohydrates, and other minerals that, if allowed to accumulate, can serve as a growing medium for decaying microorganisms."
3. **Discussion**: The potential applications of antioxidants from agricultural by-products in the pharmaceutical industry, as well as in food and animal feed additive production, should be mentioned (not only cosmetics).
4. **Discussion**: Compare the different extraction methods used to determine high antioxidant activity.
5. **Table 1**: The publishing dates should be rearranged in either ascending or descending order.

Are the rationale for, and objectives of, the Systematic Review clearly stated?

Yes

Is the statistical analysis and its interpretation appropriate?

Not applicable

If this is a Living Systematic Review, is the ‘living’ method appropriate and is the search schedule clearly defined and justified? (‘Living Systematic Review’ or a variation of this term should be included in the title.)

Yes

Are sufficient details of the methods and analysis provided to allow replication by others?

Yes

Are the conclusions drawn adequately supported by the results presented in the review?

Yes

Reviewer Expertise:

Food contaminants, bioactive metabolites, agricultural waste, activated carbon

I confirm that I have read this submission and believe that I have an appropriate level of expertise to confirm that it is of an acceptable scientific standard.
